# The Value of Strain Elastography in Predicting Autoimmune Thyroiditis

**DOI:** 10.3390/diagnostics10110874

**Published:** 2020-10-27

**Authors:** Cristina Mihaela Cepeha, Corina Paul, Andreea Borlea, Florin Borcan, Renata Fofiu, Cristina Adriana Dehelean, Dana Stoian

**Affiliations:** 1 PhD School Department, Victor Babes University of Medicine and Pharmacy, 300041 Timisoara, Romania; cristina.cepeha@yahoo.com (C.M.C.); renata.fofiu@yahoo.com (R.F.); 2 Department of Internal Medicine II, Victor Babes University of Medicine and Pharmacy, 300041 Timisoara, Romania; stoian.dana@umft.ro; 3 Department of Pediatrics, Victor Babes University of Medicine and Pharmacy, 300041 Timisoara, Romania; 4 Faculty of Pharmacy, Victor Babes University of Medicine and Pharmacy, 300041 Timisoara, Romania; fborcan@umft.ro (F.B.); cadehelean@umft.ro (C.A.D.)

**Keywords:** Hashimoto, elastography, thyroid stiffness, autoimmune disease, strain ratio

## Abstract

Chronic autoimmune thyroiditis (CAT) defines a diffuse intrathyroidal lymphocytic infiltration associating a destructive process of the thyroid follicles, most commonly in evolution developing hypothyroidism. Typical ultrasound changes may suggest the presence of the disease. This study aims to evaluate the performance of strain elastography in detecting autoimmune thyroiditis as an additional tool to the conventional ultrasound examination. A total of 250 patients were enrolled in the study; 180 had biochemical confirmation of CAT, the other 70 healthy subjects represented the control group. All patients were examined clinically and by means of conventional thyroid ultrasound (US) and real-time elastography using a Hitachi Preirus machine (5–15 MHz linear probe). Five valid measurements for the parenchyma/muscle strain ratios (SR) were taken for each subject, considering the mean value for analysis. A mean SR value above 1.64 was found to predict the presence of CAT with sensitivity (Sen) 69%, specificity p92%, positive predictive value (PPV) 95.4%, negative predictive value (NPV) 54% and area under receiver operating characteristic (AUROC) 0.87. Moreover, when comparing the mean values for SR, significantly higher values were found in CAT patients compared with the controls (2.81 ± 2.11 vs. 1.03 ± 0.51; *p* < 0.0001). Of the 180 CAT subjects, 92 were on thyroid hormone replacement therapy; significantly higher values were detected for patients under therapy compared with asymptomatic cases (3.45 ± 2.53 vs. 2.15 ± 1.27, *p* < 0.0001). A cut-off value of 2.94 was established for identifying CAT patients who needed hormonal treatment (Sen 52.3%, Sp 83.7%, PPV 75.4%, NPV 64.7% and AUROC 0.66). No correlation was found between stiffness and antibody titers nor for functional status. Elastography does add valuable information to the US evaluation of cases with autoimmune thyroiditis.

## 1. Introduction

Chronic autoimmune thyroiditis (CAT) is represented by nonspecific, chronic and irreversible inflammation of the thyroid, defined by the presence of specific antibodies against the thyroid structure, which are anti-thyroid peroxidase (ATPO) antibodies and antithyroglobulin (ATG) antibodies. As described by the histologic examination, the disease is characterized by various degrees of fibrosis along with the presence of lymphoid follicles or atrophic follicles with a small quantity of colloid containing Hurtle cells. Currently, lymphocytic chronic thyroiditis represents one of the most frequent causes of hypothyroidism in Europe, being its major determinant in iodine-replete regions [1,2]. The presence of an increased titer of anti-thyroid antibodies is described in up to 10–12% of the general population with asymptomatic cases prevailing [3,4]. The prevalence of the disease is greater in the female population and Caucasian individuals, increasing with age and sex steroids fluctuations [3,5,6]. Important evidence also supports that a certain genetic susceptibility has a clear role in autoimmune thyroid disease; family clusters and frequent association in cases with Down’s or Turner’s syndrome were observed [7,8].

The most effective technique for thyroid morphology examination is by conventional ultrasound (US); it is easily applicable, widely available and well-tolerable for the patient [9]. Diffuse thyroid disease can be diagnosed based on classical ultrasound; the challenge resides in differentiating Hashimoto’s thyroiditis from autoimmune hyperthyroidism [10]. Typical US changes may suggest the diagnosis. The classical CAT ultrasound picture illustrates hypoechogenicity, inhomogeneity and pseudonodules induced by their infiltrative pathophysiology basis [11].

In recent years, a new technology has been developed, elastography, which allows the measurement of tissue elasticity. There are two major types of elastography, strain elastography (SE) and shear-wave elastography (SWE). SE uses manual external compression or internal physiologic movements in order to measure strain while SWE measures the speed of machine-generated shear waves in order to quantify tissue elasticity [12,13]. Most often, the stiffness measurements are used in order to help differentiate malignant focal lesions from benign ones, with excellent performance. SE measures the elasticity of the tissue based on a color map with green meaning soft tissue and blue and red representing higher stiffness. The examiner exerts a slight repetitive pressure on the transducer and then chooses two regions of interest (ROI). These are healthy lesion and parenchyma in the case of nodular pathology or thyroid parenchyma and the sternocleidomastoid muscle in the case of diffuse pathology. If possible, the two ROI are placed at the same depth so that the strain ratio measurements will not be influenced by a deeper position [14]. Consecutively, the device calculates the strain ratio. Normally, the thyroid parenchyma has a homogeneous, soft appearance [12,13,14,15,16].

There are numerous studies evaluating nodular thyroid disease, which found that strain elastography has a high sensitivity (92%) and specificity (90%) for the detection of malignant thyroid nodules. SE proved helpful in reducing unnecessary fine-needle aspiration biopsies, being a useful tool in the risk stratification of thyroid nodular lesions [16,17,18,19]. However, recent data are emerging, suggesting the value of elastography in the diagnostic of diffuse thyroid disease [20] especially for SWE techniques [21,22,23,24], with very few studies involving SE [25,26,27].

Therefore, the aim of this study is to analyze the usefulness of strain elastography in predicting the presence and progression of autoimmune thyroid disease.

## 2. Materials and Methods

### 2.1. Patients

This prospective study was conducted from July 2019 to July 2020 on patients with diffuse thyroid pathology. Informed written consent was obtained from all subjects. The study was performed in accordance with the Ethics Guidelines of the Helsinki Declaration and was approved by the local Ethics Committee (“Dr. D Medical Center”, CECS nr. 09/03.06.2019).

Two hundred and fifty subjects were evaluated; 180 of them presented chronic autoimmune thyroiditis diagnosed by significant increased values of ATPO and/or ATG antibodies (10 males and 170 females; age range 18–72). The control group consisted of 70 healthy subjects (4 males and 66 females; age range 19–68) with normal thyroid function, anti-thyroid antibodies titers and ultrasound aspect.

### 2.2. Inclusion Criteria

Only patients with a certain diagnosis of CAT were included in the study. The diagnosis was suggested by clinical examination and/or ultrasound appearance and confirmed by significantly high levels of ATPO and/or ATG antibodies. The control group included subjects with healthy thyroid status, implying normal ATPO and ATG antibody levels and normal thyrotropin (TSH) and thyroxin (FT4) values. All subjects were euthyroid. Out of 180 patients diagnosed with CAT, 92 were euthyroid under supplemental treatment.

### 2.3. Exclusion Criteria

Patients presenting nodular thyroid pathology, Graves’ disease (GD) and known thyroid malignancies were excluded. Cases with a history of lobectomy or subtotal thyroidectomy were also excluded from the study group. We did not take into consideration cases that presented a suggestive ultrasound aspect for CAT but had normal anti-thyroid antibodies titers.

### 2.4. Biochemical Assay

The following measurements were taken into consideration. Thyroid-stimulating hormone (TSH) (reference range 0.27–40.2 μUI/mL; method immunochemistry with enzyme chemiluminescence immunoassay (ECLIA)), free-thyroxine (FT4) (reference range 12–22 pmol/L; method ECLIA), ATPO (reference range < 34 UI/mL; method microparticle-based chemiluminescence immunochemistry (CMIA)) and ATG (reference range < 115 UI/mL; method ECLIA).

### 2.5. Conventional Ultrasound and Elastography Examination

Conventional B-mode thyroid ultrasound and strain elastography (real-time elastography (RTE)) were performed on a Hitachi Preirus machine with a 5–15 multifrequency linear probe. All subjects were evaluated by clinical examination and ultrasonography by the same practitioner with over 15 years of experience in thyroid ultrasound. The optimal position of examination was the supine position with the head tilted back in order to fully expose the neck. Patients were asked not to swallow or talk during the examination. The thyroid was examined using grey-scale ultrasound measuring transversal (two dimensions) and longitudinal (one dimension) diameters, thyroid volume and echogenicity.

**Figure 1:**
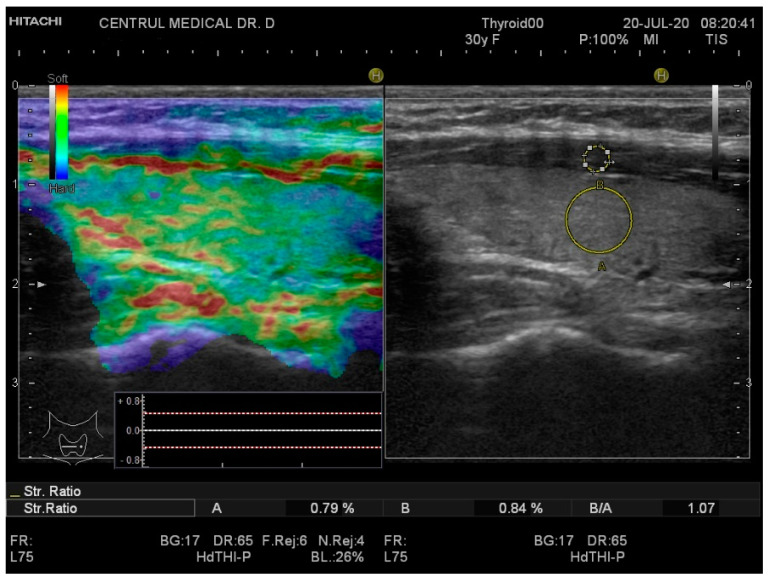
Strain elastography (**left**) and grey-scale ultrasound (US) (**right**) of a healthy patient—left thyroid lobe. The yellow circle A is the local region of interest (ROI) on the thyroid tissue; the yellow circle B is the local ROI on the sternocleidomastoid muscle adjacent to the thyroid. Strain ratio (SR) = 1.07.

**Figure 2:**
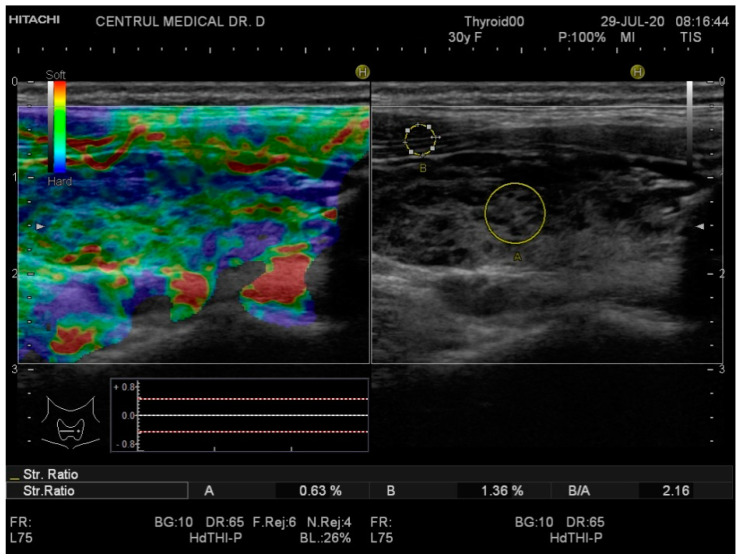
Strain elastography (**left**) and conventional US (**right**) of a patient diagnosed with chronic autoimmune thyroiditis (CAT)—right thyroid lobe. The yellow circle A is the local ROI on the thyroid tissue; the yellow circle B is the local ROI on the adjacent sternocleidomastoid muscle. SR = 2.16.

Real-time elastography was performed after conventional US during the same visit. The stiffness measurements were collected in the same session as the grey-scale US evaluation. The probe was placed perpendicularly to the skin and repetitive, light compression was applied avoiding lateral movement. All images were obtained in the longitudinal plane. A blue-green-red color map was displayed with blue indicating no strain (high stiffness), green indicating intermediate stiffness and red representing soft tissue. In order to calculate the strain ratio (SR), two regions of interest (ROI) were placed consequently. The first one, ROI A, was the thyroid tissue while the second one, ROI B, was the sternocleidomastoid muscle in front of the ipsilateral thyroid parenchyma. Figure 1 and Figure 2 below illustrate the SE image and calculation of the SR in normal thyroid tissue and, respectively, in a patient with CAT. Five consecutive measurements were made for each lobe and the mean value was considered in the final evaluation. The SR was calculated and displayed automatically for each lobe. 

### 2.6. Statistical Analysis

The statistical analysis was performed using MedCalc Software, version 12.5.0.0 (MedCalc program, Belgium) and SPSS, version 17.0 (IBM Statistics). The Kolmogorov–Smirnov test was used for testing the distribution of the numerical variables. The mean value and standard deviation were calculated for the numerical variables with normal distribution, while in cases of non-normal distribution, median values and range intervals were used. Categorical variables were reported as the number (proportion) of patients with/without the specific characteristic.

The Student’s *t*-test was used for group comparisons of continuous variables with a normal distribution and nonparametric tests (the Mann–Whitney U-test) for variables with a non-normal distribution. Group comparisons of categorical variables were performed using Pearson’s χ^2^-test.

Univariate regression analysis and multivariate regression analysis were used in order to identify the factors associated with the presence of AITD and to formulate a score for the prediction of the disease.

Areas under receiver operating characteristic (AUROC) curves were calculated for thyroid stiffness (TS) values to identify discriminating cut-offs. The optimal cut-off values were determined from the AUROC curve analysis and the Youden J index and its associated criterion values were chosen. The sensitivity (Sen), specificity (Sp), positive predictive value (PPV) (true positive cases/all positive cases), negative predictive value (NPV) (true negative cases/all negative cases) and diagnostic accuracy (sum of true positive and true negative cases/total number of cases) were calculated. Ninety-five percent confidence intervals were calculated for each predictive test and a *p*-value < 0.05 was considered to reveal statistical significance.

## 3. Results

Reliable thyroid elastography measurements using strain elastography were obtained in 250 subjects (100%); therefore, all 250 subjects were included in the final analysis. The main characteristics of the subjects included are summarized in Table 1. Numerical variables with a normal distribution are presented as a mean value ± standard deviation while variables with a non-normal distribution are presented as median values and range intervals.

**Figure 3:**
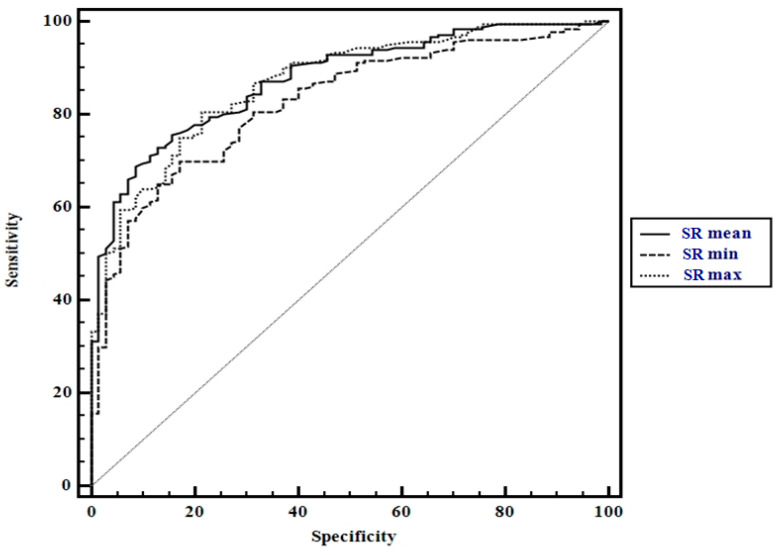
Comparison between receiver operating characteristic for SR values using mean SR values (SR mean), the highest SR values (SR max), and the lowest SR values (SR min).

Mean strain ratio (SR mean) values expressing thyroid stiffness were used for establishing the optimal cut-off for predicting CAT: SR > 1.64 (AUROC 0.87, Sen 69%, Sp 92%, PPV 95.4%, NPV 54%). When the highest SR (SR max) values were used, we obtained the following cut-off value for predicting CAT: SR > 1.9 (AUROC 0.80, Sen 70.5%, Sp 75%, PPV 78.8%, NPV 54.5%) while when the lowest SR (SR min) values were used, the following cut-off value was obtained: SR > 1.1 (AUROC 0.75, Sen 69.4%, Sp 67.5%, PPV 82.4%, NPV 52.9%) (Figure 3).

The difference between the prediction accuracies is summarized in Table 2. The value of the area below AUROC as close as possible to 1 gives the test a better predictive value. As it can be seen in Figure 3 and in Table 2, the SR mean had the best predictive value followed by SR max and then SR min. For this reason, in the following determinations we will use as standard the value SR mean.

**Figure 4:**
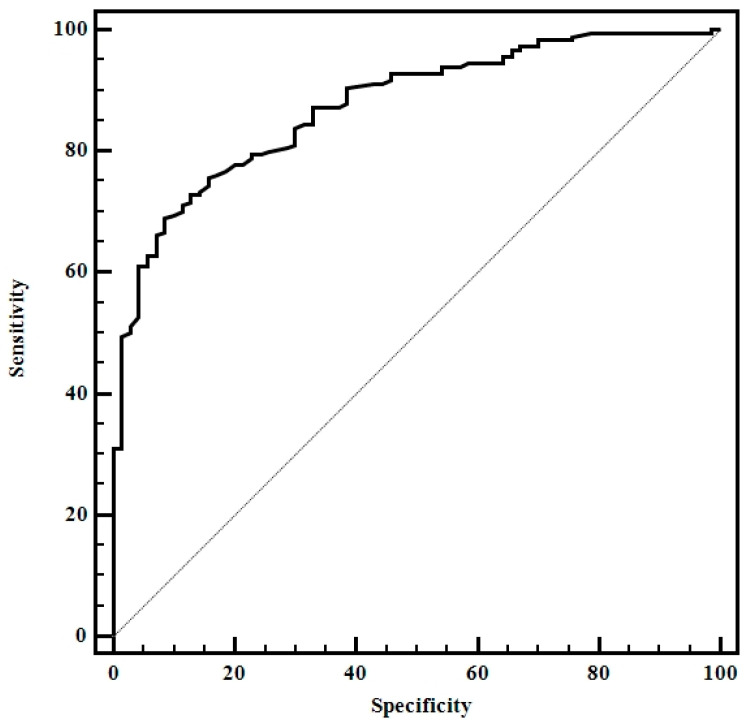
Receiver operating characteristic for thyroid stiffness.

The mean thyroid stiffness values were significantly higher for patients with CAT compared with the healthy group (2.81 ± 2.11 vs. 1.03 ± 0.51; *p* < 0.0001).The optimal cut-off value (highest sum of sensitivity and specificity) for predicting the presence of CAT was >1.64 (AUROC 0.87, Sen 69%, Sp 92%, PPV 95.4%, NPV 54%) (Figure 4).

The subjects were divided according to their age into the following subgroups: 18–20 years: 3/180; 21–30 years: 41/180; 31–40 years: 46/180; 41–50 years: 40/180; 51–60 years: 34/180; 61–70 years: 13/180; 71–80 years: 3/180. The comparison between the mean SR values of the subjects from each subgroup and the mean SR values in normal subjects is summarized in Table 3.

Significantly higher values can be observed in CAT patients compared with healthy ones in all age groups under 60 years old.

**Figure 5:**
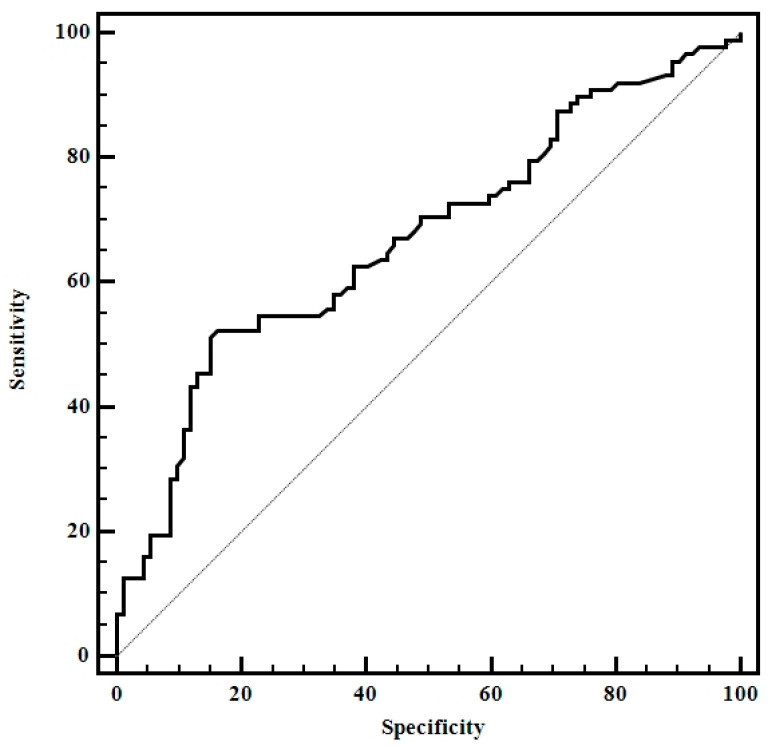
Receiver operating characteristics for thyroid stiffness for predicting hypothyroidism in CAT patients (patients under hormone replacement therapy).

Out of the 180 patients diagnosed with CAT, 92 (51.1%) were already in the hypothyroidism phase, being on thyroid hormone replacement therapy at the time of examination. Mean thyroid stiffness values were significantly higher for patients with hypothyroidism compared with those with an asymptomatic disease (3.45 ± 2.53 vs. 2.15 ± 1.27, *p* < 0.0001). An optimal cut-off value (highest sum of SE and SP) of > 2.94 was established for predicting the presence of hypothyroid status in the case of CAT patients with Sen (52.3%), Sp (83.7%), PPV (75.4%), NPV (64.7%) and AUROC (0.66) (Figure 5).

No correlation was found between thyroid stiffness values and ATPO levels (r = 0.014) nor between thyroid stiffness values and the volume of the thyroid (r = 0.053).

Age, thyroid stiffness values, anti-TPO levels and thyroid volume were tested in a univariate regression analysis as independent predictors for CAT. Only anti-TPO levels (*p* < 0.0001) and thyroid stiffness values (*p* < 0.0001) were independent predictors of CAT.

**Figure 6:**
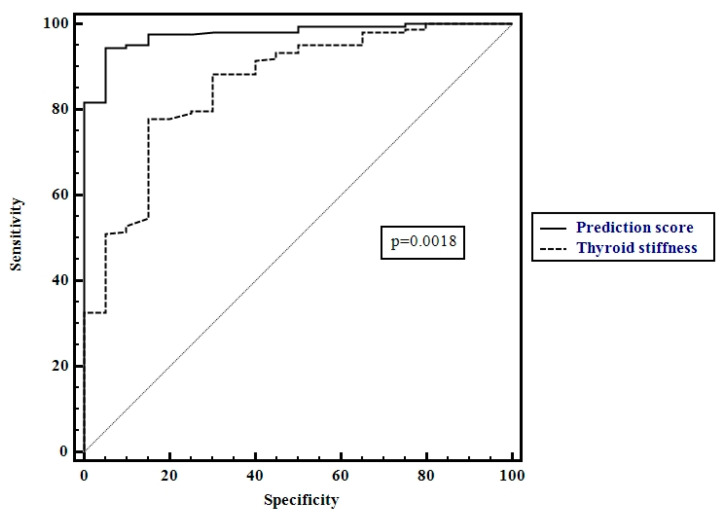
Comparison between receiver operating characteristics for thyroid stiffness values and the prediction score.

Using these factors as predictors, by multiple regression analysis we obtained the following score for predicting CAT: 0.03x thyroid stiffness values +0.x anti-TPO values +0.64. The optimal cut-off value of our score for predicting CAT was >0.7 (AUROC 0.97, Sen 95%, Sp 95%, PPV 99%, NPV 68%, *p* < 0.001). By comparing the AUROCs, the score performed better than thyroid stiffness values alone for predicting the presence of CAT (*p* = 0.0018) (Figure 6).

## 4. Discussion

Conventional ultrasound is widely used in thyroid examination, being the elective means of diagnosis. However, in the case of chronic autoimmune thyroiditis, classical ultrasound can only suggest the diagnosis that must be afterwards confirmed by laboratory tests. As previously mentioned, the histological substrate of chronic autoimmune thyroiditis is represented by lymphocytic infiltration. The interstitium presents varying degrees of fibrosis, which gives the thyroid a firm consistency. Thyrocyte lesions vary in intensity in different parts of the parenchyma, resulting in inhomogeneity and different degrees of fibrosis [28].

Thus, knowing that the inflammation increases the level of fibrosis resulting in stiffer tissue, we can investigate the inverse hypothesis, which is whether increased stiffness may describe inflammation. Most studies on thyroid elasticity have used the SWE technique and significant differences were determined between CAT and normal thyroid parenchyma.

Sedlackova et al. compared the thyroid stiffness in 46 patients with diffuse thyroid pathology and 128 healthy patients, finding lower mean and maximal stiffness in the healthy control group [29]. Similarly, another study that included 57 subjects showed differences in thyroid elasticity levels in patients with diffuse pathology compared with the control group but the differences were not statistically significant (*p* = 0.802 and *p* = 0.452) [30]. Kara et al. also found significant differences between groups but, in addition, a positive correlation between SWE measurements and both ATPO and ATG antibodies was noted as well as a significant negative association between SWE and echogenicity [21].

Very good PPV (92%) and NPV (81%) were determined in one study performed in Turkey on 50 patients diagnosed with CAT and 40 healthy subjects, which also found significant differences between the SWE measurements of the two groups [31].

Although there are numerous studies discussing the value of SWE in the diagnosis of chronic autoimmune thyroiditis, RTE was studied in only a few papers but with promising results [25,32].

Therefore, in this prospective study, strain elastography was used in order to analyze the differences in the thyroid parenchyma elasticity of subjects diagnosed with chronic autoimmune thyroiditis compared with a healthy thyroid. Similar to current literature, our results showed significant differences between the average values of the strain ratio of patients with thyroiditis compared with the controls [25,33]. It has been shown that the level of stiffness of various diffuse thyroid pathologies can be ranked in ascending order as follows: control group < hyperthyroidism group < Hashimoto’s thyroiditis group < subacute thyroiditis group [25].

Furthermore, one study that evaluated elasticity scores in various diffuse thyroid pathologies found significant differences between the levels of fibrosis of the experimental groups. They detected statistically significant SRs for the differential diagnosis of subacute thyroiditis (SAT) from GD and Hashimoto’s thyroiditis (HT) and reconfirmed that strain elastography could be a useful tool for distinguishing SAT cases from healthy ones or from other types of autoimmune disorders, respectively GD and HT [33].

Following the statistical analysis, we established the best cut-off value of 1.64 with a specificity of 92% and sensitivity of 69%. In a study including 31 patients diagnosed with CAT and 21 healthy subjects, the optimal cut-off was 0.677 (Sen 96% and Sp 67%) [26]. A different study conducted on 76 patients and 46 healthy subjects achieved a cut-off value of 0.98 (83% sensitivity and 93% specificity) but it is worth mentioning that the study population consisted of adolescents, not adults [34]. Therefore, in order to establish the optimal cut-off value for the diagnosis of chronic autoimmune thyroiditis, studies on larger populations are needed and several values may be considered for differences in populations and various US machines.

In accordance with other studies, no correlation was found between thyroid stiffness and TSH values [26]. We also determined no correlations between tissue elasticity and the age or gender of the patients. To our knowledge, there are no other data in the literature regarding this correlation.

An interesting remark that could benefit from further study is the correlation between thyroid stiffness and the titer of anti-thyroid antibodies. Our analysis did not find any correlation between these two. Menzilcioglu et al. described a positive correlation between ATPO antibodies and strain index ratio (r = 0.68) while another study concluded that no correlation was found between the level of ATG antibodies and SR but found a positive correlation between SR and ATPO antibodies (r = 0.43) [26,34]. A study conducted on a larger number of patients also concluded that SWE is useful in the diagnosis of CAT but without finding a correlation between the levels of fibrosis and ATG antibody titers (*p* = 0.101) and only a weak positive correlation between SWE and ATPO antibodies (Spearman’s correlation coefficient = 0.311) [35].

Another notable comment about the study population is that about half of the patients in the CAT group were on hormone replacement therapy with levothyroxine. In this respect, significantly higher stiffness was observed in the treatment group compared with euthyroid CAT subjects.

Elastography is a standardized method for assessing liver stiffness [36,37,38]; the vast literature confirms the very good predictions of elasticity measurements with biopsy results. There are also good studies evaluating renal stiffness in chronic kidney disease [39,40]. As mentioned, autoimmune thyroid disease by definition implies lymphocytic inflammatory infiltration and autoimmune thyroidal damage involving both the interstitium and the follicles [28,41]. Considering that chronic inflammation progresses to various degrees of fibrosis [28,42] the question that emerges is whether elastography can predict, as in diffuse liver or kidney disease, the degree of functional impairment. Knowing that CAT progresses to hypothyroid status, requiring hormone replacement therapy, the important levels of fibrosis in the group undergoing treatment may be explained by a longer course of the disease. This difference in thyroid stiffness between euthyroid and hypothyroid patients has been investigated before using SWE with similar results. A study conducted by Magri showed significantly increased stiffness in patients requiring LT4 substitution compared with those who did not (27.3 ± 9 kPa vs. 20.9 ± 10.4 kPa; *p* = 0.02) [43]. Therefore, we initiate the hypothesis of establishing a cut-off value for identifying patients who require LT4 therapy depending on the level of fibrosis measured with the use of RTE. We propose a cut-off value of >2.94 for predicting CAT with Sen 52.3%, Sp 83.7%, PPV 75.4%, NPV 64.7% and AUROC 0.66. This allows us to rule out the patients who do not reach hypothyroid status. More detailed studies are needed in this direction.

The large number of subjects included in the study as well as the novelty of investigating the differences in elasticity and functional status represent the strengths of our paper. The proposal of a prediction score for the diagnosis of CAT also brings a new diagnostic approach. A weak point of our research may be the lack of age-matched groups.

## 5. Conclusions

Thyroid elastography is currently used mostly as an additional valuable tool for determining the malignancy risk of thyroid focal lesions. Our results do support the good quality information that elastography also brings to the evaluation of cases with diffuse thyroid pathology. A correlation between antibody titers and stiffness values was not demonstrated, nor for the thyroid functional status and fibrosis but this needs further study. RTE seems to be a good predictor for detecting autoimmune thyroiditis and could therefore improve its US diagnosis.

## Figures and Tables

**Table 1:** Main characteristics of the study group (N: number of subjects; CAT: chronic autoimmune thyroiditis).

Parameter	
N	250
Age (median value and range interval)	40.8 (18–72)
Gender (%):	
Male	N = 14/250 (5.6%)
Female	N = 236/250 (94.4%)
Thyroid volume (mean ± SD)	12.05 ± 5.32
CAT	N = 180/250 (72%)
Normal subjects	N = 70/250 (28%)

For each subject, SR was measured for both thyroid lobes resulting in one minimum SR (SR min) and maximum SR (SR max). Afterwards, the average SR (SR mean) was calculated.

**Table 2:** The difference between areas under receiver operating characteristics (AUROCs) for the established cut-off value.

Parameter	AUROC	*p*-Value
Mean SR values cut-off vs. Max SR values cut-off	0.87 vs. 0.8	*p* = 0.116
Max SR values cut-off vs. Min SR values cut-off	0.8 vs. 0.75	*p* = 0.076
Mean SR values cut-off vs.Min SR values cut-off	0.87 vs. 0.75	*p* = 0.01

**Table 3:** Comparison between the mean SR values of subjects with CAT and the mean SR values in normal subjects.

SR Mean Values According to Age Subgroups		SR Mean Values in Normal Subjects		*p*-Value
18–20 years: 3/180	1.44 ± 0.17	2/70	0.58 ± 0.12	*p* = 0.009
21–30 years: 41/180	2.46 ± 1.63	20/70	1.1 ± 0.61	*p* = 0.0007
31–40 years: 46/180	2.52 ± 1.6	21/70	1.01 ± 0.52	*p* = 0.0001
41–50 years: 40/180	3.38 ± 3.06	18/70	1.05 ± 0.41	*p* = 0.0022
51–60 years: 34/180	3.06 ± 1.95	5/70	1.09 ± 0.38	*p* = 0.032
61–70 years: 13/180	3.1 ± 2.03	4/70	1.08 ± 0.73	*p* = 0.07
71–80 years: 3/180	2.15 ± 0.07			
